# A Comparison Study of Chaihu Shugan San and Fluoxetine on Antidepression and Regulating Blood Rheology Effects with Chronic Restrained Stress Rats

**DOI:** 10.1155/2020/6426383

**Published:** 2020-11-12

**Authors:** Meng Qian, Rongyan Peng, Chen Yue, Zongchun Yang, Haoru Zhu, Biyuan Liu, Ming Xie

**Affiliations:** ^1^Department of Science of Herbal Prescription, Beijing University of Chinese Medicine, Beijing, China; ^2^Dongzhimen Hospital, Beijing University of Chinese Medicine, Beijing, China; ^3^College of Chinese Medicine, Chongqing Medical University, Chongqing, China; ^4^School of Life Sciences, Beijing University of Chinese Medicine, Beijing, China

## Abstract

Chaihu Shugan San (CHSGS) is a traditional Chinese herbal formula that is often used in clinical practice to treat live Qi stagnation syndrome and depression. Fluoxetine is one of the commonly used drugs for the clinical treatment of depression. This study involved a comparison of CHSGS and fluoxetine on antidepression and regulating blood rheology effects with chronic restraint stress- (CRS-) induced depression rat models. Rats were induced depression models by CRS for 4 weeks. Upon successful induction of depression in the rats, the animal was administered CHSGS at 0.6 g/kg/d, 1.2 g/kg/d, or fluoxetine 1.8 mg/kg/d to corresponding groups by gavage for 2 weeks. The changes of CRS rats were determined by behavior observations and sucrose preference test and hypothalamic-pituitary-adrenal cortex (HPA) axis functional status. The changes in monoamine neurotransmitters and related indicators of blood status were detected by enzyme-linked immunosorbent assay (ELISA), blood rheometer, and other methods. The outcome shows that CHSGS is superior to fluoxetine in regulating the appearance and HPA axis function of model rats. In addition, CHSGS and fluoxetine have similar effects in improving blood rheology, and both can alleviate the hypercoagulable state of blood via the platelet 5-hydroxytryptamine receptor 2A (5-HT2A) pathway in rats of depression. It was also observed that CHSGS can improve the blood state of depressed rats by restoring liver coagulation-anticoagulation balance and endothelium-related functions.

## 1. Introduction

Depression is a mental disorder, which is closely related to chronic diseases such as stroke [1], cardiovascular disease [2], and diabetes [3]. Studies have pointed out that depression and adverse cardiac events have the same underlying pathological mechanism, including neuroendocrine dysfunction, cardiac autonomic control disorder, endothelial dysfunction, inflammation, and enhanced platelet reactivity [4]. In addition, the American Heart Association issued in 2014 stated that depression is closely related to the incidence rate and mortality of acute coronary syndrome [5].

Clinical studies show that the activation and aggregation rates of fibrinogen and platelet in patients with depression are significantly increased [6–8], and the levels of fibrinogen and platelet activation are related to the severity of depression [6, 7]. Among the patients with severe anxiety and depression, the levels of blood factor VII, von Willebrand factor, prothrombin fragment 1 + 2, and plasminogen activator inhibitor-1 (PAI-1) are significantly higher than those of the healthy control group [9]. Experimental studies also show that CRS enhances the platelet agonist thrombin levels and the ability of adenosine diphosphate (ADP) to stimulate platelet aggregation in mice [10]. Previous research indicated that depression has hemorheological changes characterized by hypercoagulability, which is easy to form thrombus, and is an important factor inducing cardiovascular and cerebrovascular diseases. Therefore, it is of great significance for the rational use of drugs and the prevention and treatment of cardiovascular diseases to comprehensively assess the pharmacological actions of antidepressants, especially the effects on blood rheology.

Liver Qi stagnation syndrome is one of the most common clinical syndromes in traditional Chinese medicine (TCM). Its formation is related to the negative psychological stress states such as emotional depression [11]. The clinical symptoms include depression, moodiness, chest distention and stuffiness, irritability, and the tendency of crying. It is very similar to modern depression [12]. It is also observed that the animal model of liver Qi stagnation syndrome in TCM established by CRS has behavioral characteristics similar to depression [13]. It is suggested that liver Qi stagnation syndrome in TCM might have the same pathophysiological connotation as depression in western medicine.

CHSGS is composed of *Chaihu*, *Chenpi*, *Chuanxiong*, *Xiangfu*, *Zhiqiao*, *Shaoyao*, and *Gancao*, and is the representative formula in the treatment of liver Qi stagnation syndrome by improving the depression state of liver Qi stagnation syndrome [14]. Fluoxetine is a selective serotonin reuptake inhibitor, which is widely used in the treatment of depression [15]. However, there are few studies about the effect of fluoxetine on the blood rheology of depression.

In summary, depression has the hemorheological basis of cardiovascular disease, and both Chinese herbal formula CHSGS and fluoxetine have a certain effect on treating liver Qi stagnation syndrome and depression. However, there is no report about the efficacy characteristics of the two drugs in the treatment of depression. Based on this, we made a hypothesis that “CHSGS can regulate blood rheology while improving depression.” To validate this hypothesis, this study intends to use a CRS rat model to compare CHSGS and fluoxetine on antidepression and improving blood rheology. This study provides a certain experimental basis for understanding the pathophysiological basis of the liver Qi depression syndrome in TCM and depression in western medicine, by rationally assessing the antidepression effects of the two drugs as well as their potential application values in preventing cardiovascular diseases.

## 2. Materials and Methods

### 2.1. Ethical Approval

Wistar male rats, purchased from the Beijing Vital River Laboratory Animal Technologies Co. Ltd., were maintained in a specific pathogen-free (SPF) laboratory. The experiments performed herein were approved by the Ethics Committee of Beijing University of Chinese Medicine (No. BUCM-4-2018060415-2019). In order to abide by the 3R Principle of Animal Experiments, the number of rats was minimized so that the smallest number was experimentally used while still retaining statistical significance. Thus, we chose *n* = 10 for each group.

### 2.2. Materials

CHSGS (*Chaihu*, *Chenpi*, *Chuanxiong*, *Xiangfu*, *Zhiqiao*, *Shaoyao*, and *Gancao*) was purchased from Beijing Tongrentang Technology Development Co. (Beijing, China). Fluoxetine hydrochloride capsules were purchased from Eli Lilly Suzhou Pharmaceutical Co., Ltd. (Jiangsu, China). The dopamine (DA), 5-hydroxytryptamine (5-HT), corticotropin-releasing hormone (CRH), noradrenaline (NE), corticotropin (CORT), 6-keto-protagiandinF1*α* (6-Keto-PGF1*α*), protein C (PC), free protein S (FPS), antithrombin III (AT-III), tissue-type plasminogen activator (t-PA), PAI-I, P-selectin (Ps), and thromboxane B2 (TXB2) ELISA kit were purchased from Beijing Rigorbio Science Development Co., Ltd. (Beijing, China). 5-HT2A, serotonin transporter (SERT), and glutamine transaminase (tGase) ELISA kit were purchased from Beijing Sino-UK Institute of Technology (Beijing, China). Rat calcium fluorescent probe Fluo-3 was purchased from Beijing Solarbio Science & Technology Co., Ltd. (Beijing, China).

### 2.3. Experimental Design and Tissue Collection

Male Wistar rats (*n* = 50, 9 weeks old), with an initial weight of 210–220 g, were randomly divided into 5 groups, namely, the control group, the model group, the CHSGS + 0.6 g/kg group, the CHSGS + 1.2 g/kg group, and the fluoxetine group. Except for the control group, the other groups were induced depression models by CRS [16], which is placing the rats in a cylinder of 25 cm length, 7 cm outer diameter, and 5 cm inner diameter, with a plexiglass restraint of adjustable length, for 3 h (8:00–11:00 am) daily for 28 days. During the last 2 weeks of CRS, the CHSGS + 0.6 g/kg group and the CHSGS + 1.2 g/kg group underwent intragastric administration of 0.6 g/kg/d and 1.2 g/kg/d CHSGS (CHSGS was made into granules by water extraction and alcohol precipitation, making sure each gram of granules contains 5 g crude medicine. The associated dose was determined using the “dose conversion coefficient table for animal and human body weight,” which were 2 times and 1 time of the equivalent dose, respectively). The fluoxetine group underwent an intragastric administration of 1.8 mg/kg/d. The associated dose was determined using the “dose conversion coefficient table for animal and human body weight”. The control group and the model group were given normal saline gavage during those 2 weeks. After 4 weeks, rats were sacrificed by intraperitoneal injection of 1% pentobarbital, blood and hypothalamus were taken, plasma and platelets were separated from the blood, and the hypothalamus was preserved at 80°C for detection.

### 2.4. Detection of Indicators

#### 2.4.1. Behavioral Observations [17]

The rats in each group were monitored twice a week (Tuesday and Saturday) over body weight and general behaviors (behavioral state, activity level, emotional response, and sleep state). The scores of the changes in appearance and behavior of rats in each group were counted every week, and the mean of absolute values of observations twice a week was taken. Finally, the appearance representations of similar nature were combined into two indicator groups (active state, emotional-sleep), and the scores of each indicator group were counted separately. See Table 1 for specific scoring criteria.

#### 2.4.2. Sucrose Preference Test [18]

For this test, rats were trained to consume 1% sucrose solution for 2 days prior to the start of the experiment. On the first day, the rats were trained to adapt to two bottles of 1% sucrose solution, and 24 hours later, one of the bottles was replaced with water for 24 hours. After the adaption, the rats were deprived of water and food for 24 hours. The sucrose preference test was conducted at 9:00 a.m. in which rats were individually housed, and then each was given one bottle of water and one bottle of sucrose for 1 h. On the 28th day of the modeling, the volumes of consumed sucrose solution and water were measured, and the sucrose preference was calculated by the following formula: sucrose preference = sucrose consumption/(water consumption + sucrose consumption) × 100% and was corrected for body weight.

#### 2.4.3. ELISA

The plasma samples were tested on the levels of 5-HT, CORT, Ps, TXB2, 6-keto-PGF1*α*, PC, FPS, AT-III, t-PA, and PAI-I, using specific ELISA kits according to the manufacturer's instructions. The hypothalamus samples were tested on the levels of CRH, DA, and NE, using specific ELISA kits according to the manufacturer's instructions. The platelet samples were tested on the levels of 5-HT2A, SERT, and tGase, using specific ELISA kits according to the manufacturer's instructions.

#### 2.4.4. Ca^2+^ Concentration Detection

First, an equal volume of 20% Pluronic F127 solution was added to Fluo-3, AM/DMSO solution. Second, the sample was added to Fluo-3, AM working solution, and incubated at 37°C for 20 minutes. Then, 5 times the volume of HBSS containing 1% fetal bovine serum was added and continued to incubate for 40 minutes. Next, the cells were washed 3 times with HEPES buffer saline. Then, the cells were resuspended with HEPES buffer saline to make a solution of 1 × 10^5^ cells/mL, incubated at 37°C for 10 minutes, and then used for detection. The excitation wavelength was 506 nm, and the emission wavelength was 526 nm.

#### 2.4.5. Other Indicators

Whole blood viscosity, whole blood reduced viscosity, plasma viscosity, erythrocyte sedimentation rate, and erythrocyte sedimentation equation *K* value were detected by a blood rheometer. Platelet aggregation rate and the level of plasma coagulated with four items, prothrombin time (PT), activated partial thromboplastin time (APTT), thrombin time (TT), and fibrinogen (FIB), were detected by an automatic coagulation instrument.

### 2.5. Statistical Analysis

SPSS statistics 20.0 software was used for statistical analyses. All of the data presented were the mean ± standard deviation (SD) and were compared by one-way ANOVA with data at a normal distribution. Nonnormal distribution data were analyzed using nonparametric statistics. *p* value <0.05 was considered to be statistically significant.

## 3. Results

### 3.1. Effects of CHSGS and Fluoxetine on the Appearance and Behavior in CRS Rats

**Figure 1:**
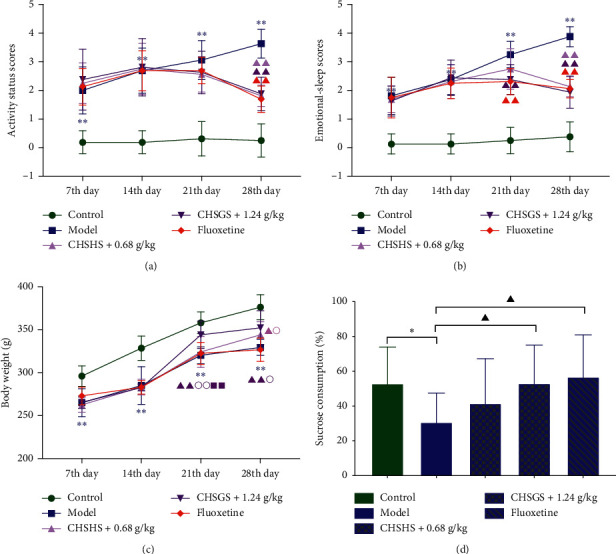
Effects of CHSGS and fluoxetine on the appearance and behavior in CRS rats. (a) Activity status scores. (b) Emotional-sleep scores. (c) Body weight. (d) Sucrose consumption. All data were expressed as the mean ± SD, *n* = 8, ^*∗∗*^*p* < 0.01, ^*∗*^*p* < 0.05, compared with the control group; ^▲▲^*p* < 0.01, ^▲^*p* < 0.05, compared with the model group; ^○○^*p* < 0.01, ^○^*p* < 0.05, compared with the fluoxetine group; ^■■^*p* < 0.01, ^■^*p* < 0.05, compared with the CHSGS + 0.6 g/kg group.

There was no significant change in the activity status scores and the emotional-sleep scores of the control group at different time points in the experiment (Figures 1(a) and 1(b)). The activity status scores were significantly increased in the model group at 1–4 weeks compared with those of the control group (*p* < 0.01) (Figure 1(a)). The activity status scores were significantly decreased in the CHSGS + 0.6 g/kg group and the CHSGS + 1.2 g/kg group at the 4th week compared with those of the model group (*p* < 0.01) (Figure 1(a)). The emotional-sleep scores were significantly increased in the model group at 1–4 weeks compared with those of the control group (*p* < 0.01) (Figure 1(b)). The emotional-sleep scores were significantly decreased in the CHSGS + 1.2 g/kg group and the fluoxetine group in the 3rd week and significantly decreased in the CHSGS + 0.6 g/kg group, the CHSGS + 1.2 g/kg group, and the fluoxetine group in the 4th week compared with those of the model group (*p* < 0.01) (Figure 1(b)). The body weight was decreased significantly from week 1 to week 4 in the model group compared with that of the control group (*p* < 0.01) (Figure 1(c)). The body weight was increased significantly in the 3rd week and 4th week in the CHSGS + 1.2 g/kg group and significantly increased in the 3rd week in the CHSGS + 0.6 g/kg group compared with that of the model group (*p* < 0.01 or <0.05) (Figure 1(c)). The body weight was increased significantly in the 3rd week in the CHSGS + 0.6 g/kg group and significantly increased in the 3rd week and 4th week in the CHSGS + 1.2 g/kg group compared with that of the fluoxetine group (*p* < 0.01 or <0.05) (Figure 1(c)). The body weight was increased significantly in the 3rd week in the CHSGS + 1.2 g/kg group compared with that of the CHSGS + 0.6 g/kg group (*p* < 0.01) (Figure 1(c)). The sucrose consumption was significantly decreased in the model group compared with that of the control group (*p* < 0.05) (Figure 1(d)). The sucrose consumption was significantly increased in the CHSGS + 1.2 g/kg group and the fluoxetine group compared with that of the model group (*p* < 0.05) (Figure 1(d)).

### 3.2. Effects of CHSGS and Fluoxetine on the HPA Axis in CRS Rats

**Figure 2:**
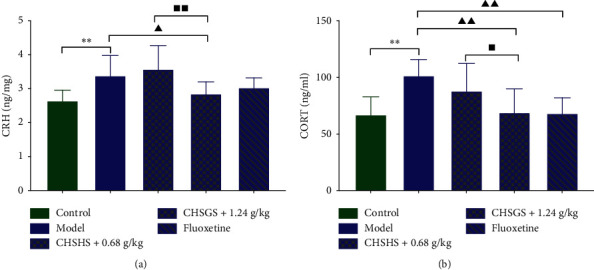
Effects of CHSGS and fluoxetine on the HPA axis in CRS rats. (a) The CRH levels in the hypothalamus. (b) The CORT levels in plasma. All data were expressed as the mean ± SD, *n* = 8, ^*∗∗*^*p* < 0.01, compared with the control group; ^▲▲^*p* < 0.01, ^▲^*p* < 0.05, compared with the model group; ^■■^*p* < 0.01, ^■^*p* < 0.05, compared with the CHSGS + 0.6 g/kg group.

The CRH levels in the hypothalamus and CORT levels in plasma were significantly increased in the model group compared with those of the control group (*p* < 0.01) (Figures 2(a) and 2(b)). The CRH levels in the hypothalamus and CORT levels in plasma were significantly decreased in the CHSGS + 1.2 g/kg group compared with those of the model group (*p* < 0.01 or 0.05) (Figures 2(a) and 2(b)). The CORT levels in plasma were significantly decreased in the fluoxetine group compared with those of the model group (*p* < 0.01 or 0.05) (Figure 2(b)). The CRH levels in the hypothalamus and CORT levels in plasma were significantly decreased in the CHSGS + 1.2 g/kg group compared with those of the CHSGS + 0.6 g/kg group (*p* < 0.01 or 0.05) (Figures 2(a) and 2(b)).

### 3.3. Effects of CHSGS and Fluoxetine on the Monoamine Neurotransmitter in CRS Rats

**Figure 3:**
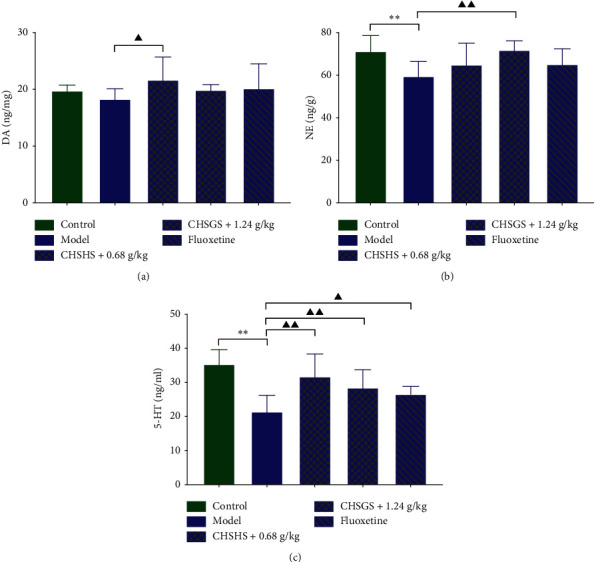
Effects of CHSGS and fluoxetine on the monoamine neurotransmitter in CRS rats. (a) The DA levels in the hypothalamus. (b) The NE levels in the hypothalamus. (c) The 5-HT levels in plasma. All data were expressed as the mean ± SD, *n* = 8, ^*∗∗*^*p* < 0.01, compared with the control group; ^▲▲^*p* < 0.01, ^▲^*p* < 0.05, compared with the model group.

The NE levels in the hypothalamus and 5-HT levels in plasma were significantly decreased in the model group compared with those of the control group (*p* < 0.01) (Figures 3(b) and 3(c)). The DA levels in the hypothalamus were significantly increased in the CHSGS + 0.6 g/kg group compared with those of the model group (*p* < 0.05) (Figure 3(a)). The NE levels in the hypothalamus were significantly increased in the CHSGS + 1.2 g/kg group compared with those of the model group (*p* < 0.05) (Figure 3(b)). The 5-HT levels in plasma were significantly increased in the CHSGS + 0.6 g/kg group, the CHSGS + 1.2 g/kg group, and the fluoxetine group compared with those of the model group (*p* < 0.01 or 0.05) (Figure 3(c)).

### 3.4. Effects of CHSGS and Fluoxetine on Blood Rheology in CRS Rats

**Figure 4:**
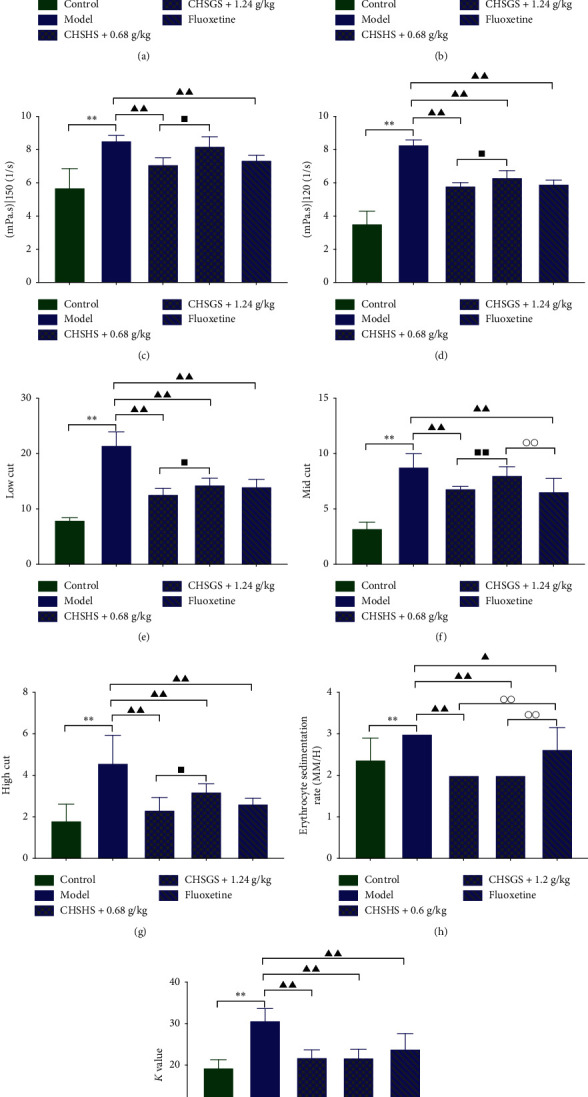
Effects of CHSGS and fluoxetine on blood rheology in CRS rats. (a) Low-cut whole blood viscosity. (b) Middle-cut whole blood viscosity. (c) High-cut whole blood viscosity. (d) The plasma viscosity. (e) Low-cut whole blood reduced viscosity. (f) Middle-cut whole blood reduced viscosity. (g) High-cut whole blood reduced viscosity. (h) Erythrocyte sedimentation rate. (i) Blood sedimentation equation *K* value. All data were expressed as the mean ± SD, *n* = 8, ^*∗∗*^*p* < 0.01, compared with the control group; ^▲▲^*p* < 0.01, ^▲^*p* < 0.05, compared with the model group; ^○○^*p* < 0.01, ^○^*p* < 0.05, compared with the fluoxetine group; ^■■^*p* < 0.01, ^■^*p* < 0.05, compared with the CHSGS + 0.6 g/kg group.

The plasma viscosity, the whole blood viscosity of low cut, medium cut, and high cut, the whole blood reduced viscosity of low cut, medium cut, and high cut, the blood sedimentation rate, and the blood sedimentation equation *K* value were significantly increased in the model group compared with those of the control group (*p* < 0.01 or 0.05) (Figure 4). The plasma viscosity, the whole blood viscosity of low cut and medium cut, the whole blood reduced viscosity of low cut, medium cut, and high cut, the blood sedimentation rate, and the blood sedimentation equation *K* value were significantly decreased in the CHSGS + 0.6 g/kg group compared with those of the model group (*p* < 0.01 or 0.05) (Figures 4(a), 4(b), and 4(d)–4(i)). The plasma viscosity, the whole blood viscosity of low cut, medium cut, and high cut, the whole blood reduced viscosity of low cut and high cut, the blood sedimentation rate, and the blood sedimentation equation *K* value were significantly decreased in the CHSGS + 1.2 g/kg group compared with those of the model group (*p* < 0.01 or 0.05) (Figures 4(a)–4(e) and 4(g)–4(i)). The plasma viscosity, the whole blood viscosity of low cut, medium cut, and high cut, the whole blood reduced viscosity of low cut, medium cut, and high cut, the blood sedimentation rate, and the blood sedimentation equation *K* value were significantly decreased in the fluoxetine group compared with those of the model group (*p* < 0.01 or 0.05) (Figure 4). The whole blood viscosity of low cut and the whole blood reduced viscosity of medium cut were significantly increased and the erythrocyte sedimentation rate was significantly decreased in the CHSGS + 1.2 g/kg group compared with those of the fluoxetine group (*p* < 0.01 or 0.05) (Figures 4(a), 4(f), and 4(h)). The erythrocyte sedimentation rate was significantly decreased in the CHSGS + 0.6 g/kg group compared with that of the fluoxetine group (*p* < 0.01) (Figure 4(h)). The plasma viscosity, the whole blood viscosity of low cut and high cut, and the whole blood reduced viscosity of low cut, medium cut, and high cut were significantly increased compared with those of the CHSGS + 0.6 g/kg group (*p* < 0.05) (Figures 4(b)–4(g)).

### 3.5. Effects of CHSGS and Fluoxetine on Four Items of Coagulation in CRS Rats

**Figure 5:**
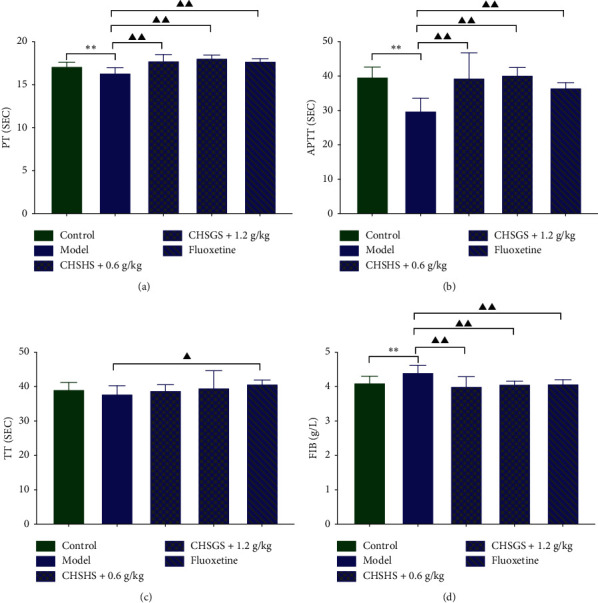
Effects of CHSGS and fluoxetine on four items of coagulation in CRS rats: (a) PT; (b) APTT; (c) TT; (d) FIB level in blood. All data were expressed as the mean ± SD, *n* = 8, ^*∗∗*^*p* < 0.01, compared with the control group; ^▲▲^*p* < 0.01, ^▲^*p* < 0.05, compared with the model group.

The PT and APTT were significantly decreased and the FIB was significantly increased in the model group compared with those of the control group (*p* < 0.01 or 0.05) (Figures 5(a), 5(b), and 5(d)). The PT and APTT were significantly increased and the FIB was significantly decreased in the CHSGS + 0.6 g/kg group, the CHSGS + 1.2 g/kg group, and the fluoxetine group compared with those of the model group (*p* < 0.01) (Figures 5(a), 5(b), and 5(d)). The TT was significantly increased in the fluoxetine group compared with that of the model group (*p* < 0.05) (Figure 5(c)).

### 3.6. Effects of CHSGS and Fluoxetine on Platelet Aggregation Rate in CRS Rats

**Figure 6:**
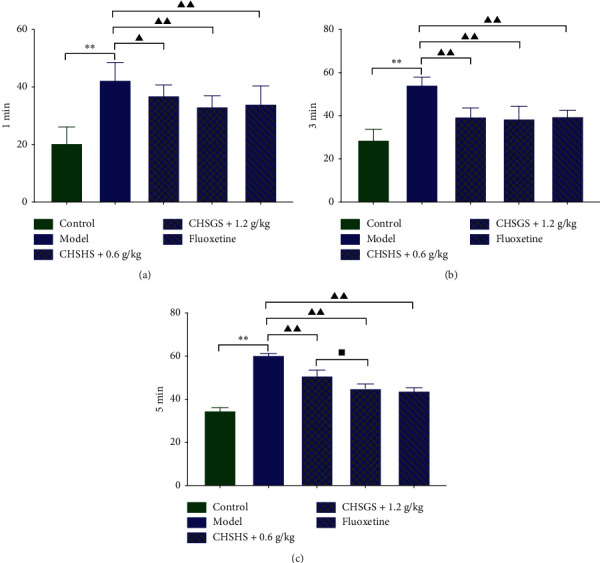
Effects of CHSGS and fluoxetine on platelet aggregation rate in CRS rats: (a) platelet aggregation rate at 1 min; (b) platelet aggregation rate at 3 min; (c) platelet aggregation rate at 5 min. All data were expressed as the mean ± SD, *n* = 8, ^*∗∗*^*p* < 0.01, compared with the control group; ^▲▲^*p* < 0.01, ^▲^*p* < 0.05, compared with the model group; ^■^*p* < 0.05, compared with the CHSGS + 0.6 g/kg group.

The platelet aggregation rates at various time points were significantly increased in the model group compared with those of the control group (*p* < 0.01) (Figure 6). The platelet aggregation rates at various time points were significantly decreased in the CHSGS + 0.6 g/kg group, the CHSGS + 1.2 g/kg group, and the fluoxetine group compared with those of the model group (*p* < 0.01) (Figure 6). The platelet aggregation rates at 5 min were significantly decreased in the CHSGS + 1.2 g/kg group compared with those of the CHSGS + 0.6 g/kg group (*p* < 0.05) (Figure 6(c)).

### 3.7. Effects of CHSGS and Fluoxetine on the 5-HT2A Signaling Pathway in CRS Rats

**Figure 7:**
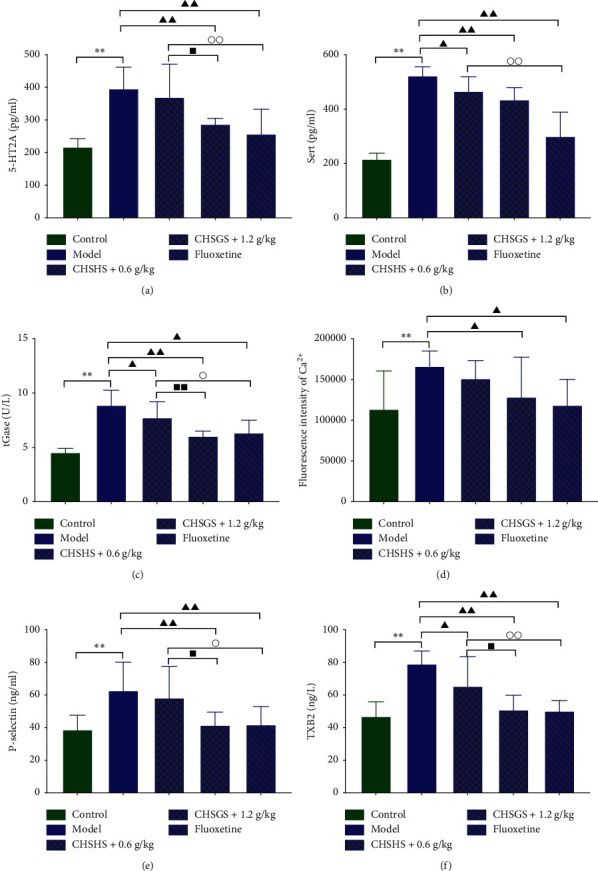
Effects of CHSGS and fluoxetine on the 5-HT2A signaling pathway in CRS rats: (a) 5-HT2A levels in platelets; (b) SERT levels in platelets; (c) tGase levels in platelets; (d) fluorescence intensity of Ca^2+^; (e) Ps levels in plasma; (f) TXB2 levels in plasma. All data were expressed as the mean ± SD, *n* = 8, ^*∗∗*^*p* < 0.01, compared with the control group; ^▲▲^*p* < 0.01, ^▲^*p* < 0.05, compared with the model group; ^○○^*p* < 0.01, ^○^*p* < 0.05, compared with the fluoxetine group; ^■■^*p* < 0.01, ^■^*p* < 0.05, compared with the CHSGS + 0.6 g/kg group.

The 5-HT2A, SERT, tGase, and Ca^2+^ levels in platelets and the Ps and TXB2 levels in plasma were significantly increased in the model group compared with those of the control group (*p* < 0.01) (Figure 7). The 5-HT2A, SERT, tGase, and Ca^2+^ levels in platelets and the Ps and TXB2 levels in plasma were significantly decreased in the CHSGS + 1.2 g/kg group and the fluoxetine group compared with those of the model group (*p* < 0.01 or 0.05) (Figure 7). The SERT tGase levels in platelets and the TXB2 levels in plasma were significantly decreased in the CHSGS + 0.6 g/kg group compared with those of the model group (*p* < 0.05) (Figures 7(b), 7(c), and 7(f)). The 5-HT2A, SERT, and tGase levels in platelets and the Ps and TXB2 levels in plasma were significantly increased in the CHSGS + 0.6 g/kg group compared with those of the fluoxetine group (*p* < 0.01 or 0.05) (Figures 7(a)–7(c), 7(e), and 7(f)). The SERT tGase levels in platelets and the Ps and TXB2 levels in plasma were significantly decreased in the CHSGS + 1.2 g/kg group compared with those of the CHSGS + 0.6 g/kg group (*p* < 0.01 or 0.05) (Figures 7(a), 7(b), 7(e), and 7(f)).

### 3.8. Effects of CHSGS and Fluoxetine on the Anticoagulant Cofactor in CRS Rats

**Figure 8:**
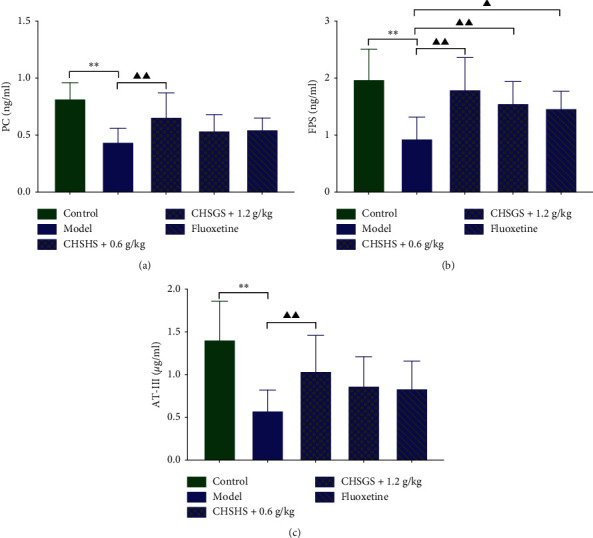
Effects of CHSGS and fluoxetine on the anticoagulant cofactor in CRS rats. (a) PC levels in plasma. (b) FPS levels in plasma. (c) AT-III levels in plasma. All data were expressed as the mean ± SD, *n* = 8, ^*∗∗*^*p* < 0.01, compared with the control group; ^▲▲^*p* < 0.01, ^▲^*p* < 0.05, compared with the model group.

The PC, FPS, and AT-III levels in plasma were significantly decreased in the model group compared with those of the control group (*p* < 0.01) (Figure 8). The PC, FPS, and AT-III levels in plasma were significantly increased in the CHSGS + 0.6 g/kg group compared with those of the model group (*p* < 0.01) (Figure 8). The FPS levels in plasma were significantly increased in the CHSGS + 1.2 g/kg group and the fluoxetine group compared with those of the model group (*p* < 0.01 or 0.05) (Figure 8(b)).

### 3.9. Effects of CHSGS and Fluoxetine on the Fibrinolysis Cofactor in CRS Rats

**Figure 9:**
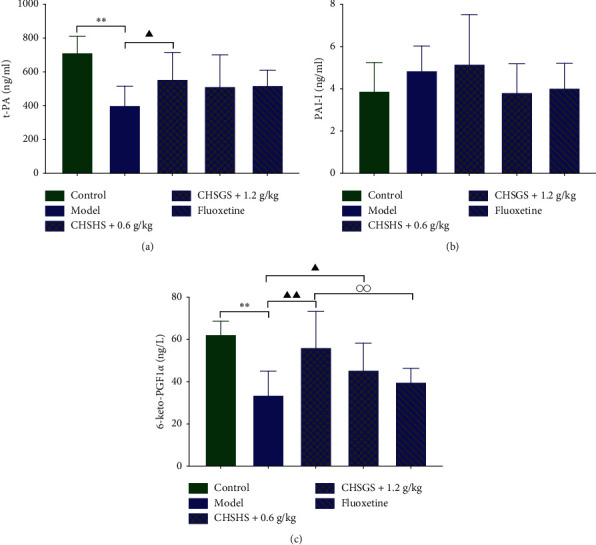
Effects of CHSGS and fluoxetine on the fibrinolysis cofactor in CRS rats. (a) t-PA levels in plasma. (b) 6-keto-PGF1*α* levels in plasma. (c) PAI-I levels in plasma. All data were expressed as the mean ± SD, *n* = 8, ^*∗∗*^*p* < 0.01, compared with the control group; ^▲▲^*p* < 0.01, ^▲^*p* < 0.05, compared with the model group; ^○○^*p* < 0.01, compared with the fluoxetine group.

The t-PA and 6-keto-PGF1*α* levels in plasma were significantly decreased in the model group compared with those of the control group (*p* < 0.01) (Figures 9(a) and 9(c)). The t-PA and 6-keto-PGF1*α* levels in plasma were significantly increased in the CHSGS + 0.6 g/kg group compared with those of the model group (*p* < 0.01 or 0.05) (Figures 9(a) and 9(c)). The 6-keto-PGF1*α* levels in plasma were significantly increased in the CHSGS + 1.2 g/kg group compared with those of the model group (*p* < 0.05) (Figure 9(c)). The 6-keto-PGF1*α* levels in plasma were significantly increased in the CHSGS + 0.6 g/kg group compared with those of the fluoxetine group (*p* < 0.01) (Figure 9(c)).

## 4. Discussion

### 4.1. Establishment of Liver Qi Stagnation Syndrome/Depression Rat Model

Although depression and liver Qi stagnation syndrome belong to different medical systems, clinical and experimental studies have shown that both of them have monoamine neurotransmitter disturbance [19, 20], HPA axis dysfunction [21, 22], and abnormal blood rheology [23, 24]. It is suggested that depression and liver Qi stagnation syndrome have a common pathophysiological basis. At present, there are 9 replication types of depression animal models, including learned helplessness (LH), unpredictable chronic mild stress (UCMS), early-life stress model, and CRS model [25]. Among them, the CRS model is relatively simple and has been widely used in the research of depression. It is also a recognized model of liver Qi stagnation syndrome in TCM [26]. Therefore, this study chose CRS to establish the rat model of liver Qi stagnation syndrome and depression.

In this experiment, we observed the appearance changes of model rats after two weeks of modeling, such as less movement, slow response, and loss of appetite and weight. After four weeks of modeling, sucrose consumption decreased significantly. It was consistent with previous experimental results [27], indicating that the model rats were in a depressed state. It was also observed that the levels of CRH in the hypothalamus and CORT in plasma were significantly increased, and the levels of NE in the hypothalamus and 5-HT in plasma were significantly decreased in model rats. It indicates that the model rats have peripheral monoamine neurotransmitter reduction and HPA axis hyperfunction, which is consistent with the neuroendocrine changes of depression [28].

### 4.2. The Antidepressant Effect of CHSGS and Fluoxetine

CHSGS is an effective compound formula for clinical treatment of liver Qi stagnation syndrome [14] and depression [29] in TCM. Fluoxetine is one of the commonly used medicines for depression in western medicine [17]. Pharmacological studies have shown that, as a nontricyclic antidepressant, it mainly inhibits the reuptake of 5-HT in the central nervous system and prolongs and improves the effect of 5-HT in order to treat depression [16]. This study observed the effect of the two drugs on the appearance behavior and neuroendocrine system of the model and compared the utility results of them. See Tables 2 and 3.

Both Tables 2 and 3 illustrate that CHSGS and fluoxetine improved the appearance behavior and laboratory indicators of CRS rats in varying degrees after two weeks of administration. Among them, the CHSGS + 1.2 g/kg group had a more obvious effect than the CHSGS + 0.6 g/kg group. Both CHSGS and fluoxetine had a positive effect on the appearance behavior score, sucrose consumption, and monoamine transmitter of model rats. CHSGS could also antagonize the weight loss and HPA axis hyperfunction of model rats, while fluoxetine had no obvious effect. It is suggested that CHSGS is superior to fluoxetine in the comprehensive neuroendocrine regulation of the depression model rats.

### 4.3. Changes of Blood Coagulation and Rheology in CRS Rats

This experiment explored the blood state of depression model rats from three aspects: blood rheology, changes in blood coagulation time, and platelet activation and aggregation. Blood rheology examination mainly reflects the changes of blood fluidity, stagnant, and viscosity [30]. In four items of coagulation, PT, APTT, TT, and FIB are included. PT and APTT reflect the level of the coagulation factor in plasma. TT is the time required for fibrinogen to be converted into fibrin, reflecting the presence of anticoagulants and fibrinolysis in plasma. FIB is the precursor of fibrin, which reflects the content of fibrinogen in the blood. Fibrinogen can be enzymatically converted into fibrin by thrombin, which blocks blood vessels and prevents excessive bleeding. The content of FIB indicates whether the body is in a hypercoagulable state [31]. The platelet aggregation test mainly reflects the aggregation of platelets, and its intensity is closely related to blood hypercoagulability.

In this experiment, it was observed that the low-cut, middle-cut, and high-cut whole blood viscosity and whole blood reduced viscosity, plasma viscosity, erythrocyte sedimentation rate, and blood sedimentation equation *K* value of CRS rats were significantly increased. PT and APTT were significantly decreased, while FIB and platelet aggregation rate at 1 min/3 min/5 min were significantly increased. This indicates that the blood rheology disorder of model rats involved the enhancement of coagulation, the decrease of fibrinolysis, and the enhancement of platelet aggregation, speculating that the blood is in a hypercoagulable state and has a tendency of thrombosis.

### 4.4. Regulatory Effect of CHSGS and Fluoxetine on Blood Rheology in CRS Rats

#### 4.4.1. Positive Effect on Blood Rheology of Model Rats

The effect of CHSGS and fluoxetine on blood rheology of model rats was compared. See Tables 4 and 5.

Both Tables 4 and 5 illustrate that both CHSGS and fluoxetine groups have a significant improvement in blood state-related indicators. The utility spectrum of CHSGS and fluoxetine was similar. For example, they could improve blood rheology indicators, prolong blood coagulation time, reduce blood fibrinogen content, and inhibit platelet activation and aggregation. The results showed that both of them could improve the hypercoagulable state of blood in CRS rats. Apart from it, CHSGS seemed to be superior to fluoxetine in reducing erythrocyte sedimentation rate, while fluoxetine could prolong TT, suggesting that the anticoagulant mechanism of fluoxetine might involve the activation of fibrinogen.

Previous studies showed that CHSGS significantly reduced the blood viscosity and platelet aggregation rate of the liver Qi stagnation syndrome rat model by CRS [13]. Fluoxetine could significantly reduce the whole blood viscosity [32] and platelet aggregation and activity [33] in patients with depression. This study found that CHSGS and fluoxetine could not only improve blood rheology and inhibit platelet aggregation but also regulate the abnormalities of the coagulation/fibrinolysis system associated with depression. It is suggested that CHSGS and fluoxetine could improve blood rheology and reduce thrombosis of depressed patients in multiple ways.

#### 4.4.2. Mechanism of CHSGS and Fluoxetine on Blood Rheology in CRS Rats

*(1) Mechanism of Abnormal Blood Rheology in Depression Model Rats*. Platelet activation plays an important role in the coagulation process. 5-HT can promote platelet aggregation as a weak platelet agonist [34]. 99% of 5-HT in the body is stored in the dense granules of platelets. When the body is depressed, the 5-HT receptor on the surface of platelets is upregulated [35]. It causes excessive 5-HT and SERT transported into platelets, activates tGase, increases Ca^2+^ concentration, and then induces the release of a large number of coagulation cofactors, such as Ps and TXA2 (TXB2 as stable metabolite), which further leads to the enhancement of platelet activation and aggregation [36–38].

Liver and vascular endothelium play a very important role in maintaining the balance of the coagulation state. The liver is not only the organ producing and secreting most clotting factors (reflected by four items of coagulation) but also can regulate the balance of coagulation and anticoagulant by producing anticoagulant auxiliary factors such as AT-III and PC and inhibit blood hypercoagulability and abnormal thrombosis. AT-III binds with activated coagulation factors and then hydrolyzes, thereby blocking the coagulation cascade. After PC activation, it combines with FPS, degrades coagulation factors Va and VIIIa, prevents their activation to form thrombin, and inhibits platelet activation. In addition, the combination of PC and FPS can inhibit the action of PAI-1, an inhibitor of plasminogen activator [39]. It is beneficial to the activation of plasminogen and inhibits thrombosis.

Endothelial cells can regulate the blood rheology through secreting factors such as anticoagulant cofactor prostacyclin (PGI2), fibrinolysis cofactor t-PA, and PAI-1. PGI2 (6-keto-PGF1*α* as its stable metabolite) can activate adenylate cyclase, synthesize cAMP, and reduce cytosolic Ca^2+^ level to inhibit platelet activation. Through activating plasminogen, t-PA is converted into plasmin and breaks down blood clots [40]. PAI-1 can inhibit the effect of t-PA, inhibit fibrinolysis, and make blood clots difficult to degrade [41].

In a word, liver and endothelial cells can maintain the coagulation-anticoagulation balance by producing coagulation, anticoagulation, and fibrinolysis cofactors, inhibiting platelet activity, and degrading and inactivating thrombin, coagulation factors, and fibrin in blood hypercoagulability.

In this experiment, 5-HT in the plasma of CRS rats was significantly decreased, expressions of platelet 5-HT2A and SERT were significantly increased, and tGase, Ca^2+^, Ps, and TXB2 in the platelets were significantly increased. It is suggested that 5-HT in the peripheral circulation of depression model rats might be overtransported and enriched in platelets. On the one hand, it reduced the level of 5-HT in the blood. On the other hand, it activated the tGase-Ca^2+^ pathway in platelets, which led to the release of a large number of coagulation cofactors, promoted further activation and aggregation of platelets, and finally led to blood hypercoagulation. It was also found that the levels of the anticoagulant cofactor (PC, FPS, and AT-III), 6-keto-PGF1*α*, and t-PA in the plasma were significantly reduced. Combined with the results of four items of coagulation (PT, APTT shortened, FIB increased), it is speculated that abnormal blood rheology of depression model rats may be related to the abnormal accumulation of 5-HT in platelets and the further activation of platelets, as well as the abnormal regulation of liver and endothelium on blood coagulation and anticoagulation/fibrinolysis cofactors.

*(2) Comparison of the Mechanism of CHSGS and Fluoxetine on Hemorheology in CRS Rats*. The mechanism of CHSGS and fluoxetine on blood rheology of CRS rats was compared. See Tables 6 and 7.

Table 6 illustrates that the high dose of CHSGS and fluoxetine could increase 5-HT in plasma, downregulate the expression of platelet 5-HT2A and SERT, and reduce the level of tGase and Ca^2+^ in platelets and the secretion of coagulation cofactors (Ps and TXB2). The results showed that both CHSGS and fluoxetine could inhibit the activation of platelets by inhibiting the accumulation of 5-HT in platelets, thereby improving the hypercoagulability of the model rats. It was found by comparison that the low dose of CHSGS had no obvious effect on platelet 5-HT2A, Ca^2+^, and Ps-selectin, or it was inferior to the high dose group. It is suggested that CHSGS might have a dosing advantage in this link. In addition, the effect of the high dose of CHSGS on platelet 5-HT2A and tGase appears to be more significant than that of fluoxetine.

Table 7 illustrates that CHSGS + 0.6 g/kg group had a regulating effect on multiple indicators of fibrinolysis/anticoagulation cofactors in CRS rats. High dose of CHSGS could increase 6-keto-PGF1*α* and FPS, while the fluoxetine group only increased FPS. The results suggested that the high and low doses of CHSGS might have selectivity while improving the blood rheology state. High dose mainly regulated the platelet system, while low dose focused on the regulation of liver and endothelium. Compared with fluoxetine, CHSGS had advantages in regulating the balance of coagulation/fibrinolysis cofactors in model rats. It is speculated that the effect of CHSGS on the blood rheology of depression might involve the regulation of the liver and endothelium. It is suggested that this formula could regulate the coagulation-anticoagulation balance and improve blood hypercoagulability, so as to reduce the risk of cardiovascular diseases in patients with depression.

### 4.5. The Effect Material Basis of CHSGS

The ingredients of CHSGS are relatively complex and include saikosaponin, paeoniflorin, hesperidin, ferulic acid, tetramethylpyrazine, and albiflorin [42]. Among them, saikosaponin, paeoniflorin, hesperidin, and albiflorin have obvious antidepressant effects [43, 44], and ferulic acid and ligustrazine have the pharmacological activity of inhibiting platelet aggregation [45, 46]. Therefore, it is speculated that the antidepressant, anticoagulant, and blood rheological effects of this formula may be related to the ingredients such as saikosaponin, hesperidin, ferulic acid, and ligustrazine in this formula.

## 5. Conclusion

The CRS rats not only showed depression-related appearance behaviors and changes in laboratory indicators but also had abnormalities in blood rheology. Further exploration showed that the CRS rats had the abnormal regulation of platelet 5-HT2A signal pathway, the abnormal expression of blood coagulation and anticoagulant/fibrinolytic cofactor which was related to liver and endothelium, and the imbalance of liver coagulation-anticoagulant relationship. It is suggested that the depression model induced by CRS could be used in mechanism research and drug evaluation of depression with the risk of cardiovascular diseases.

CHSGS in TCM and fluoxetine in modern medicine could not only improve the appearance behaviors and neuroendocrine abnormalities of the depression model rats but also improve the hypercoagulable state of blood to different degrees. The regulation effects of CHSGS on behavior observation and HPA axis hyperfunction in CRS rats were more obvious than those in fluoxetine. They had the same effect on improving blood rheology. The mechanism involved the regulation of the platelet 5-HT2A signaling pathway, but CHSGS was also related to the recovery of liver coagulation-anticoagulation balance and the regulation of endothelial function.

This study provided further evidence for the common pathophysiology basis between liver Qi stagnation syndrome in TCM and depression in modern medicine. It also provided pharmacological evidence over the potential advantages of CHSGS and fluoxetine in the treatment of depression accompanied with the risk of cardiovascular diseases.

## Figures and Tables

**Table 1:** General appearance behavior scoring standards in rats.

Observed indicators	Scoring standards (score)				
Behavioral state	Getting together 2	Less moving 1	Normal 0	Much moving 1	Restlessness 2
Activity level	Sluggish 2	Weaken 1	Normal 0	Much moving 1	Excited 2
Emotional response	Sluggish 2	Slower 1	Normal 0	Irritable 1	Angry 2
Sleep state	Lethargic 2	Tired 1	Normal 0	Light sleep 1	Easily awakened 2

**Table 2:** Comparison of the effect of CHSGS and fluoxetine on the appearance behavior of CRS rats.

Group	Body weight				Activity status scores				Emotional-sleep scores				Sucrose consumption
	1 w	2 w	3 w	4 w	1 w	2 w	3 w	4 w	1 w	2 w	3 w	4 w	
Model	↓↓	↓↓	↓↓	↓↓	↑↑	↑↑	↑↑	↑↑	↑↑	↑↑	↑↑	↑↑	↓
CHSGS + 0.6 g/kg	—	—	—	↑	—	—	—	↓↓	—	—	—	↓↓	—
CHSGS + 1.2 g/kg	—	—	↑↑	↑↑	—	—	—	↓↓	—	—	↓↓	↓↓	↑
Fluoxetine	—	—	—	—	—	—	—	↓↓	—	—	↓↓	↓↓	↑

*Note*. In the table, the model group was compared with the normal group, and others were compared with the model group. ↑ or ↓, *p* < 0.05;↑↑ or ↓↓, *p* < 0.01; — , no significant difference.

**Table 3:** Comparison of the effect of CHSGS and fluoxetine on laboratory indicators of CRS rats.

Group	Monoamine neurotransmitter			HPA axis	
	DA	NE	5-HT	CRH	CORT
Model	—	↓↓	↓↓	↑↑	↑↑
CHSGS + 0.6 g/kg	↑	—	↑↑	—	—
CHSGS + 1.2 g/kg	—	↑	↑↑	↓	↓↓
Fluoxetine	—	—	↑	—	↓↓

*Note*. In the table, the model group was compared with the normal group, and others were compared with the model group. ↑ or ↓, *p* < 0.05; ↑↑ or ↓↓, *p* < 0.01; —, no significant difference.

**Table 4:** Comparison of the effect of CHSGS and fluoxetine on blood rheology of CRS rats.

Group	Whole blood viscosity			Plasma viscosity	Erythrocyte sedimentation rate	*K* value	Whole blood reduced viscosity		
	Low cut	Middle cut	High cut				Low cut	Middle cut	High cut
Model	↑↑	↑↑	↑↑	↑↑	↑↑	↑↑	↑↑	↑↑	↑↑
CHSGS + 0.6 g/kg	↓↓	↓↓	↓↓	↓↓	↓↓	↓↓	↓↓	↓↓	↓↓
CHSGS + 1.2 g/kg	↓↓	↓↓	—	↓↓	↓↓	↓↓	↓↓	—	↓↓
Fluoxetine	↓↓	↓↓	↓↓	↓↓	↓	↓↓	↓↓	↓↓	↓↓

*Note*. In the table, the model group was compared with the normal group, and others were compared with the model group. ↑↑ or ↓↓, *p* < 0.01; —, no significant difference.

**Table 5:** Comparison of the effect of CHSGS and fluoxetine on blood coagulation of CRS rats.

Group	PT	APTT	TT	FIB	Platelet aggregation rate		
					1 min	3 min	5 min
Model	↓↓	↓↓	—	↑↑	↑↑	↑↑	↑↑
CHSGS + 0.6 g/kg	↑↑	↑↑	—	↓↓	↓	↓↓	↓↓
CHSGS + 1.2 g/kg	↑↑	↑↑	—	↓↓	↓↓	↓↓	↓↓
Fluoxetine	↑↑	↑↑	↑	↓↓	↓↓	↓↓	↓↓

*Note*. In the table, the model group was compared with the normal group, and others were compared with the model group. ↑ or ↓, *p* < 0.05; ↑↑ or ↓↓, *p* < 0.01; —, no significant difference.

**Table 6:** Comparison of CHSGS and fluoxetine on the regulation of platelet activity in CRS rats.

Group	5-HT	5-HT2A	SERT	tGase	Ca^2+^	Ps	TXB2
Model	↓↓	↑↑	↑↑	↑↑	↑↑	↑↑	↑↑
CHSGS + 0.6 g/kg	↑↑	—	↓	↓	—	—	↓
CHSGS + 1.2 g/kg	↑↑	↓↓	↓↓	↓↓	↓	↓↓	↓↓
Fluoxetine	↑	↓↓	↓↓	↓	↓	↓↓	↓↓

*Note*. In the table, the model group was compared with the normal group, and others were compared with the model group. ↑ or ↓, *p* < 0.05; ↑↑ or ↓↓, *p* < 0.01; —, no significant difference.

**Table 7:** Comparison of CHSGS and fluoxetine on the effect of the coagulation system in CRS rats.

Group	Anticoagulant				Fibrinolysis	
	6-keto-PGF1*α*	PC	FPS	AT-III	t-PA	PAI-1
Model	↓↓	↓↓	↓↓	↓↓	↓↓	—
CHSGS + 0.6 g/kg	↑↑	↑↑	↑↑	↑↑	↑	—
CHSGS + 1.2 g/kg	↑	—	↑↑	—	—	—
Fluoxetine	—	—	↑	—	—	—

*Note*. In the table, the model group was compared with the normal group, and others were compared with the model group. ↑ or ↓, *p* < 0.05; ↑↑ or ↓↓,; —, no significant difference.

## Data Availability

The data used to support the findings of this study are included within the article.

## Abbreviations

CHSGS: Chaihu Shugan San
CRS: Chronic restraint stress
DA: Dopamine
5-HT: 5-Hydroxytryptamine
CRH: Corticotropin-releasing hormone
NE: Norepinephrine
CORT: Corticotropin
6-Keto-PGF1*α*: 6-keto-protagiandinF1*α*
PC: Protein C
FPS: Free protein S
AT-III: Antithrombin III
t-PA: Tissue-type plasminogen activator
PAI-I: Plasminogen activator inhibitor type 1
Ps: P-selectin
TXB2: Thromboxane B2
5-HT2A: 5-Hydroxytryptamine receptor 2A
SERT: Serotonin transporter
tGase: Glutamine transaminase
PT: Prothrombin time
APTT: Activated partial thromboplastin time
TT: Thrombin time
FIB: Fibrinogen.
